# Oral manifestations of monkeypox and potential tools for their management

**DOI:** 10.11604/pamj.2023.45.137.40573

**Published:** 2023-07-20

**Authors:** Cinzia Casu, Martina Salvatorina Murgia, Germano Orrù

**Affiliations:** 1 Innovation Sciences and Technologies, University of Cagliari, Cagliari, Italy,; 2 Department of Surgical Science, Oral Biotechnology Laboratory, University of Cagliari, Cagliari, Italy

**Keywords:** Monkeypox, photodynamic therapy, oral pathology

## To the editors of the Pan African Medical Journal

Since May 2022, cases of monkeypox have been reported in around 70 countries where the disease is not endemic. The World Health Organization (WHO) has declared the 2022 monkeypox outbreak a Public Health Emergency of International Concern. The monkey poxvirus, like that of smallpox, is a member of the Orthopoxvirus group. There are two distinct strains (or clades, i.e., groups of similar microorganisms that are descended from a common ancestor) of monkeypox: the West African strain and the Congo Basin strain [1]. Particularly, all cases associated with the 2022 global outbreak are caused by the West African strain. Despite the name, nonhuman primates are not a reservoir for the virus. Although the reservoir is unknown, the main candidates are small rodents (e.g. squirrels) of African rainforests, mainly in western and central Africa [1]. Dentists and oral pathologists should be aware of the clinical manifestations of the new monkeypox virus. Even if this last is transmitted through large respiratory droplets and typically requires prolonged close contact (in contrast to the current SARS-CoV-2 infection), the possibility of transmission through saliva splashes during dental procedures is concrete [2]. It has also been documented by a recent work on the new health emergency, which in 43% affects the oral cavity (25%) and the perioral region in general [3]. In many cases, oral lesions may precede skin rash, fever, lymphadenopathy, and other associated major symptoms [2,3]. The few case reports currently in the literature dedicated to oral clinical manifestations report mainly erosive lesions at the lingual level, erosive-erythematous crusted at the labial level [4]. The lingual lesions described involve the part adjacent to the lingual tip in correspondence of the midline, both at the dorsal level and at the ventral level. Multiple erosive lesions, are reported and in one case generalized lingual swelling associated with burning [5]. All patients described in the case reports were male, aged between 26 and 38 years, promiscuous, some of them with a previous clinical history of HIV or syphilis. In three of them the oral manifestations were the first ever to appear, while in two cases the oral lesions appeared at the same time as the genital ones [6].

In the latter case [6], a brush performed on the erosive-crusted labial lesions excluded the presence of HSV DNA, HZV DNA, and syphilis. The possibility of carrying out tests on suspicious lingual and labial lesions by oral pathologists who, on their first visit, find suspicious lesions on these patients, should be considered as a method of early diagnosis and therefore of containment of the epidemic. No data have been reported on the duration of oral manifestations and on the specific treatment of these lesions. Given the lack of specific therapeutic strategies for the management and effective treatment of the infection caused by this virus, we are looking for non-conventional therapies. Among these, due to the absence of side effects and the ease of execution, photodynamic therapy (PDT) stands out. PDT is based on the use of a non-toxic dye called photosensitizer, which is activated at a specific wavelength resulting in the production of reactive oxygen species (ROS), which cause irreversible damage to diseased cell structures without damaging healthy ones [7,8]. In the literature, a study performed a computational strategy to investigate the potential of PDT using propoly-benzofuran against monkeypox virus demonstrating that PDT based on this natural photosensitizer can be proposed as an adjuvant treatment against this virus [9]. Furthermore, in the literature there is a case report of a man with active symptomatic smallpox virus infection with facial skin ulcerative lesions effectively treated with a combination of antimicrobial PDT and photobiomodulation therapy [10]. In this specific case, 0.01% methylene blue was used activated by a laser light at 660 nm wavelength perpendicularly and in contact mode [10].

**Conclusion:** given the lack of specific strategies for the management of oral monkeypox virus infections, current evidences in the literature suggest that PDT seems to be a promising therapeutic approach, also by virtue of the absence of side effects and the low therapeutic cost. Therefore, although more studies are needed in this regard, in the light of the results that have emerged.
